# How Many Is Enough?—Statistical Principles for Lexicostatistics

**DOI:** 10.3389/fpsyg.2016.01916

**Published:** 2016-12-12

**Authors:** Menghan Zhang, Tao Gong

**Affiliations:** ^1^Ministry of Education Key Laboratory of Contemporary Anthropology, Collaborative Innovation Center of Genetics and Development, School of Life Sciences, Fudan UniversityShanghai, China; ^2^Haskins LaboratoriesNew Haven, CT, USA; ^3^Center for Linguistics and Applied Linguistics, Guangdong University of Foreign StudiesGuangdong, China

**Keywords:** Swadesh lists, cognates, Bernoulli process, binomial distribution, Ansari-Bradley test, Spearman's rho

## Abstract

Lexicostatistics has been applied in linguistics to inform phylogenetic relations among languages. There are two important yet not well-studied parameters in this approach: the conventional size of vocabulary list to collect potentially true cognates and the minimum matching instances required to confirm a recurrent sound correspondence. Here, we derive two statistical principles from stochastic theorems to quantify these parameters. These principles validate the practice of using the Swadesh 100- and 200-word lists to indicate degree of relatedness between languages, and enable a frequency-based, dynamic threshold to detect recurrent sound correspondences. Using statistical tests, we further evaluate the generality of the Swadesh 100-word list compared to the Swadesh 200-word list and other 100-word lists sampled randomly from the Swadesh 200-word list. All these provide mathematical support for applying lexicostatistics in historical and comparative linguistics.

## Introduction

In linguistics, quantitative approaches such as lexicostatistics and glottochronology have been widely applied to detect hypothetical genetic relations among languages (McMahon and McMahon, 2005; Campbell, 2013). Lexicostatistics refers to the statistical manipulation of lexical materials for historical inferences that abstract away from exact dates (Hymes, 1960). Lexicostatistics compares languages for phylogenetic affinity based on proportion of cognates in a standard basic vocabulary list. Each slot in the list is a concept (meaning), and collected items (words) occupying the same slot are compared cross-linguistically. Some linguists suggest using the term “meaning list” instead of “word list” or “vocabulary list” because the latter two are potentially ambiguous (McMahon and McMahon, 2005). We thus do not make distinction between the terms vocabulary list and meaning list. Unlike lexicostatistics, glottochronology deals in particular with phylogenetic relationships among languages (Campbell, 2013). Strictly speaking, lexicostatistics is a broader approach than glottochronology without specific assumptions such as constant rate of word retention or loss.

Computing lexicostatistics generally proceeds in the following steps (McMahon and McMahon, 2005; Campbell, 2013):

1. *Assemble a set of word forms from languages being compared based on a list of basic vocabulary*. It would be ideal to collect every word from languages being compared, yet it is infeasible to obtain an exhaustive or very large-scale collection of words, especially for endangered or poorly-documented languages. In practice, linguists usually conduct basic word assembly based on small-scale meaning lists. Two widely-adopted lists for this purpose are the Swadesh lists. They compile 100 (Swadesh, 1955) or 200 (Swadesh, 1952) concepts. The choice of these concepts is determined mainly by linguistic intuitions and experiences. For example, it has been argued that words encoding these concepts are stable and resistant to borrowing; therefore, the chances that identified cognates are due to borrowing or contact, rather than phylogenetic relation, are reasonably low (note that about 10% of the Swadesh 100-word list are still prone to borrowing). The Swadesh lists have been employed to construct many linguistic datasets of Indo-European and Austronesian languages (e.g., Dyen et al., 1997; Lohr, 2000; Greenhill et al., 2008; Wichmann et al., 2013).
2. *Identify lexical cognates based on recurrent sound correspondences*. Cognates and recurrent sound correspondences provide strong evidence of a common origin of languages. Recurrent sound correspondences typically occur in vocabulary of languages having phylogenetic relations (or systematically borrowed words in languages having a history of deep contact; Hoijer, 1956; Bergsland and Vogt, 1962). In definition, a “recurrent” sound correspondence must occur in at least two or more matching instances. However, given that there are not many assembled words for comparison, nobody can give a satisfactory answer to questions such as how many instances in the collected words that exhibit a sound matching would allow linguists to classify it as a recurrent sound correspondence, rather than borrowing (Hoijer, 1956; Hock and Joseph, 1996). In other words, there lacks a concrete threshold, in terms of the minimum number of sound matching instances, for determining a recurrent sound correspondence. In practice, linguists often adopt an iterative approach by exhaustively (if possible) listing all matching instances across a given word list to identify a recurrent sound correspondence.

Apart from classical lexicostatistics, there exist a number of additional quantitative approaches in language comparison research, all of which follow roughly the same steps as above (e.g., Oswalt, 1971; Ringe, 1992; Baxter and Ramer, 2000; Lohr, 2000; Kessler, 2001). Given a fixed vocabulary list and a number of lexical cognates exhibiting recurrent sound correspondences, hypothetical phylogenetic relations among languages can be verified.

Despite of its wide applications, there are a number of objections to lexicostatistics (Bergsland and Vogt, 1962; Eska and Ringe, 2004; McMahon and McMahon, 2006). Many of the critics focus on the composition of the vocabulary list, such as what concepts can be utilized for collecting potential cognates and whether it is possible to construct a universal concept list for cognate assembly. Other critics concern the uncertainties inherent in the two steps above. Some of these uncertainties deserve more discussion here (Baxter and Ramer, 2000).

First, it remains unclear whether comparison among 100 or 200 Swadesh words can reasonably demonstrate the relatedness between languages. Apart from the Swadesh lists, some linguists suggest using much smaller vocabulary lists for cognate collection. Some of these lists contain 40 (Holman et al., 2008), 35 (Starostin, 2000), 33 (Baxter and Ramer, 2000), or only 15 (Dolgopolsky, 1986) concepts. By contrast, others advocate using much bigger lists for this purpose, which consist of 300–500 concepts (Greenberg, 1990; Ruhlen, 1994; Li, 1995; Newman, 1995; Huang, 1997; Jiang, 2007). Linguistic intuitions and experiences are still the primary considerations to construct these lists (Heggarty, 2010), and many lists share several concepts with the Swadesh lists.

Second, the threshold of recurrent sound correspondence is subject to not only the size of the vocabulary list but also the occurring frequencies of involved segments in the list. For example, if the vocabulary list is big and two segments appear frequently in the assembled words according to this list, the chance of finding an accidental correspondence or borrowing between them would increase. Hence, it would require more instances of such correspondence to confirm whether it is a recurrent correspondence or not. By contrast, if the vocabulary list is small and two segments are less frequent, a small number (say, two) of matching instances is sufficient to confirm recurrent correspondence between the segments (Ringe, 1992; Kessler, 2001).

Third, Swadesh argued that the 100-word list could reliably reflect the vertical, inheritance relations among languages (Swadesh, 1955). Due to the ease of gathering 100 words compared to 200, many language comparison studies have directly used the Swadesh 100-word list for word assembly. Before taking this simpler approach, one needs to clarify whether the Swadesh 100-word list is quantitatively a special sub-list of the Swadesh 200-word list. This can be clarified by the following two questions:

1. Whether the distribution of sound correspondences in the words collected by the Swadesh 100-word list can reliably resemble the distribution of the same correspondences in the words collected by the Swadesh 200-word list;
2. Whether the distribution based on the Swadesh 100-word list can resemble those based on other 100-word sub-lists constructed by randomly sampling from the Swadesh 200-word list. In other words, whether a random split of the Swadesh 200-word list into two 100-word lists yields significantly distinct distributions; if so, the Swadesh 100-word list would not be a special sub-list of the Swadesh 200-word list.

In this paper, we attempt to tackle the above uncertainties from a mathematical perspective. We propose two statistical principles to calculate, respectively, a “conventional” size of the vocabulary list to collect and judge potential cognates and a “reasonable” threshold to identify recurrent sound correspondences. Here, the “conventional” size means the most convenient and informative size of the vocabulary list especially in situations where it is not possible to collect many word forms, or where there is little prior knowledge about the languages being compared. The “reasonable” threshold means the flexible threshold at least accounting for the occurring frequencies of phonetic segments in the collected words. With more information about languages being available, the actual threshold can be updated accordingly. Apart from these principles, we also adopt some standard statistical tests to evaluate the generality of the Swadesh 100-word list. All the analyses are done in MATLAB (ver. 2015a) and based on some well-known examples. They can be reasonably extended to other complex cases.

In the following sections, we derive the statistical principles from stochastic sampling theorems, and apply them in real cases of assembling cognates and detecting recurrent correspondences. Based on the empirical data and statistical tests, we also evaluate the generality of the Swadesh 100-word list. For the sake of simplicity, we only address two-language comparison. Finally, we discuss the importance of these principles to lexicostatistics.

## Statistical principles and example results

### Conventional size of the vocabulary list to assemble potential cognates

We model the task of setting a conventional size of the basic vocabulary list for collecting potentially true cognates as a statistical task of constructing an exemplar set by sampling from a total set. Here, the total set refers to the total vocabulary *V* of a language, which contains *N* words. For the sake of simplicity, we assume that *V* in each language being compared has roughly the same size. The exemplar set *X* (⊂ *V*) contains *n* words (*x*_1_, *x*_2_, *x*_3_, …*x*_*n*_, *n* < *N*), each chosen from *V*. Under this setting, the statistical task is to determine *n*, such that the distribution of sound correspondences in *X* (obtained by comparing each pair of words aligned by semantic equivalence) approximately matches (reaching a predefined significance level α and within a predefined error rate ε) the distribution of those correspondences in *V*. In mathematical terms, such matching can be described as in Equation (1):

(1) $$\begin{array}{l} P\left(|\bar{X}-\mu |<\varepsilon \right)=1-\alpha ,\text{ where }\bar{X}=\frac{1}{n}\overset{n}{\underset{i=1}{\sum }}x_{i},\;\mu =E\left(V\right) \end{array}$$

In practice, sampling potential cognates is not random, but guided by the concepts in the adopted vocabulary list. However, in all languages, mappings between meanings and phonetic structures in word forms are largely arbitrary (Hockett, 1960; De Saussure, 1983; Chomsky, 1995; Hock and Joseph, 1996; Hurford, 2012), in the sense that apart from social convention of using word A for meaning B there is no explicit connection between the sound of a word and aspects of its meaning (Dingemanse, 2012). Note that there exist a small proportion of words that show iconicity between the form and meaning aspects (e.g., onomatopoeia, words imitating natural sounds, often in a highly language-specific way; and ideophones, words vividly evoking sensory impressions like sounds, movements, textures, visual patterns, or actions; Dingemanse, 2012; Dingemanse et al., 2015), but these words and their concepts usually do not appear in the vocabulary list for cognate assembly. Such arbitrariness allows us to reasonably simplify the process of sampling potential cognates as a random sampling process.

Strictly speaking, sampling potential cognates is conducted without replacement; after choosing a word from *V* and putting it to *X*, remove it from *V*. However, considering the much bigger size *N* of *V* than the size *n* of *X* (*N*≫*n*) and the arbitrariness in mappings between semantics and phonetics, the sampling process can be viewed as a process with replacement (still keep the word in *V* after sampling it). In this way, exemplars in *X* are independent and identically distributed (*i.i.d*.).

The above simplifications enable us to apply stochastic sampling theorems to this task. According to *the central limit theorem* (the probability distribution of the mean of *i.i.d*. variables with finite variance approximately follows a normal distribution), no matter whether the distribution of sound correspondences in *V* is known or not, the distribution of normalized sound correspondences in *X* approximates the standard normal distribution with mean 0 and standard deviation 1. In mathematical terms, this distribution can be described by Equation (2) (see Walpole et al., 2011: p. 234 for proof):

(2) $$\begin{array}{l} P\;(Z<U_{\frac{\alpha }{2}})=1-\alpha ,\text{ where }Z=\frac{\left|\bar{X}-\mu \right|}{\bar{\sigma }},\;{\bar{\sigma }}^{2}=Var\;(\bar{X}) \end{array}$$

Here, $U_{\frac{\alpha }{2}}$ is set according to significance level α (see Table 1).

**Table 1:** **$U_{\frac{\alpha }{2}}$ of the standard normal distribution at different significant level α, and the conventional sizes calculated using Equation (8) at error rate ε = 0.05 or 0.1 and total vocabulary size ***N*** = 4000 or 5000**.

**α**	**0.2**	**0.1**	**0.05**	**0.02**	**0.01**	**0.002**
$U_{\frac{\alpha }{2}}$	**1.282**	**1.645**	**1.96**	**2.326**	**2.576**	**3.09**
*n* (ε = 0.05, *N* = 4000)	157.866	253.456	350.498	476.568	569.158	770.815
*n* (ε = 0.1, *N* = 4000)	40.670	66.526	93.788	130.833	159.288	225.260
*n* (ε = 0.05, *N* = 5000)	159.122	256.709	356.750	488.202	585.829	801.713
*n* (ε = 0.1, *N* = 5000)	40.753	66.748	94.230	131.694	160.567	227.826

*Gray cells indicate the calculated conventional sizes in the eight conditions of our estimation*.

According to the statistics of random sampling with replacement, the variance of the distribution of normalized sound correspondences in *X* can be expressed by the variance of the distribution of sound correspondences in *V*, as in Equation (3) (see Walpole et al., 2011: p. 154, p. 767 for proofs):

(3) $$\begin{array}{l} {\bar{\sigma }}^{2}=Var\;(\bar{X})=\frac{\sigma^{2}}{n}\frac{N-n}{N-1}\approx \frac{\sigma^{2}}{n}\left(1-\frac{n}{N}\right),\;where\;\sigma^{2}\\ =Var\left(V\right) \end{array}$$

Linking Equation (2) with Equation (3), we have:

(4) $$\begin{array}{l} P\;(|\bar{X}-\mu |<\bar{\sigma }U_{\frac{\alpha }{2}})=P\;(|\bar{X}-\mu |<\frac{\sigma }{\sqrt{n}}U_{\frac{\alpha }{2}}\sqrt{1-\frac{n}{N}})\\ =1-\alpha \end{array}$$

Linking Equation (4) with Equation (1), we have:

(5) $$\begin{array}{l} \varepsilon =\frac{\sigma }{\sqrt{n}}U_{\frac{\alpha }{2}}\sqrt{1-\frac{n}{N}},\;then,\;n{\left(\frac{\varepsilon }{\sigma U_{\frac{\alpha }{2}}}\right)}^{2}=1-\frac{n}{N} \end{array}$$

Solving for *n* from Equation (5), yields:

(6) $$\begin{array}{l} n=\frac{N{\left(\sigma U_{\frac{\alpha }{2}}\right)}^{2}}{N\varepsilon^{2}+{\left(\sigma U_{\frac{\alpha }{2}}\right)}^{2}} \end{array}$$

**Figure 1:**
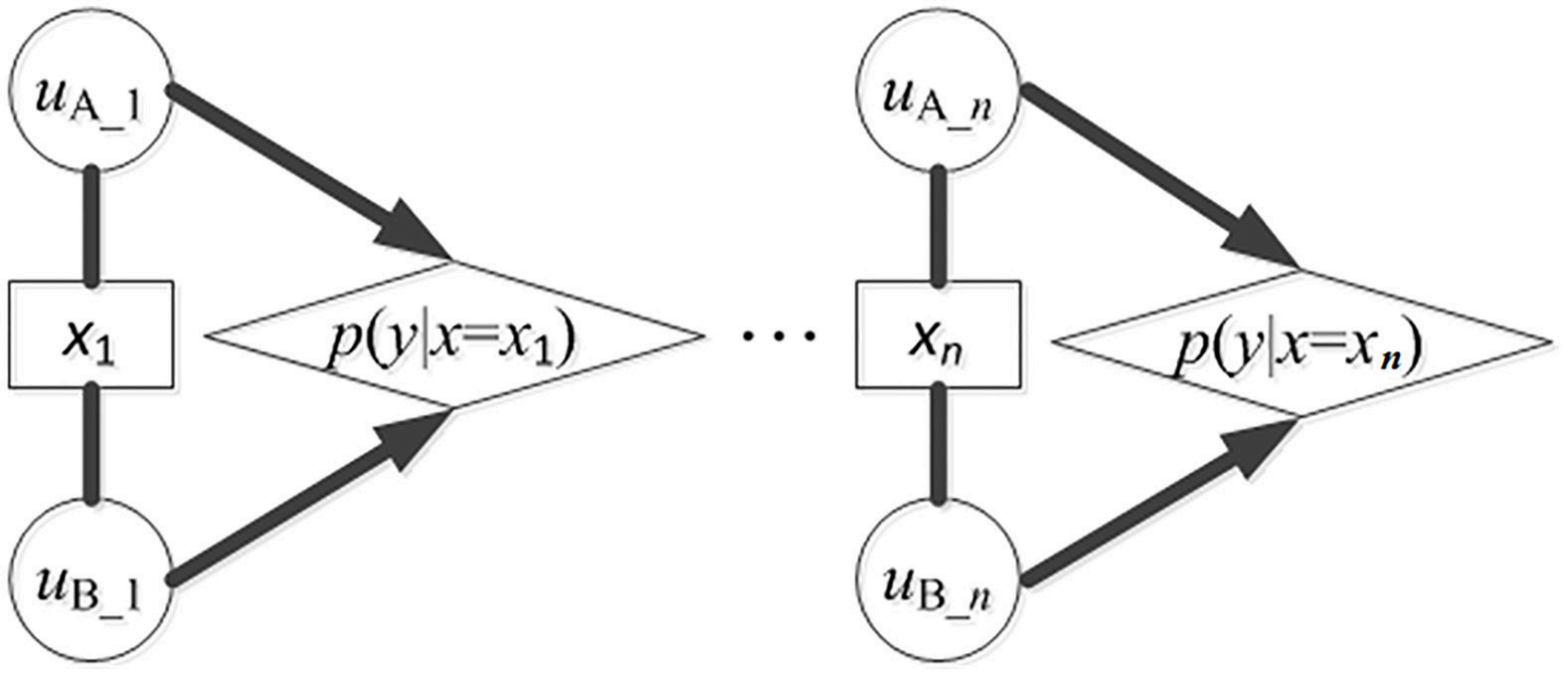
**Detection of sound correspondences in assembled words from languages A and B**. *x*_*i*_ (*i* = 1 to *n*) is the concept in the vocabulary list for collecting potential cognates, *n* is the size of the vocabulary list. *u*_*A*_*i*_ is the word form from A that is semantically equivalent to *x*_*i*_. *u*_*B*_*i*_ is the word form from B that is semantically equivalent to *x*_*i*_. *p*(*y*|*x* = *x*_*i*_) is the probability that some segments in *u*_*A*_*i*_ and *u*_*B*_*i*_ show a correspondence. The detecting process can be conceived as a Bernoulli process. The probabilities of showing correspondences in all exemplars follow a 0–1 distribution, and the probabilities for a particular correspondence to occur different times in all exemplars follow a binomial distribution.

Given a list of collected words with semantic equivalence from languages being compared, comparison between each pair of words having equivalent meanings and, respectively, from the two languages being compared represents a single trial of phonetic matching between the two languages. Such comparison has two possible outcomes: match or mismatch (see Figure 1). Note that this is obviously an initial model that glosses over problems of semantic ambiguity and one-to-many or many-to-one matches. Considering that the exemplars in *X* are independent and identically distributed (*i.i.d*.), the process of detecting sound correspondence among the exemplars can be conceived as a Bernoulli process having two outcomes (exhibiting a sound correspondence or not). Accordingly, the probability distribution of sound correspondences in *X* follows a Binomial Distribution, in which the probability of having a sound correspondence in a pair of exemplars with semantic equivalence is *p* (then, the probability of not having a sound correspondence is 1 − *p*).

Following the binomial distribution, the variance of the distribution in *V* approximates the variance of the distribution in *X*, the latter of which is calculated as in Equation (7) (see Walpole et al., 2011: p. 130 for proof):

(7) $$\begin{array}{l} \sigma^{2}=Var\;(V)\approx {\bar{\sigma }}^{2}=p\left(1-p\right) \end{array}$$

Link Equation (7) with Equation (6) and we have:

(8) $$\begin{array}{l} n=\frac{N{\left(U_{\frac{\alpha }{2}}\right)}^{2}p\left(1-p\right)}{N\varepsilon^{2}+{\left(U_{\frac{\alpha }{2}}\right)}^{2}p\left(1-p\right)} \end{array}$$

Without prior knowledge of potential sound correspondences (this is often the case when linguists face the data of a language for the first time) and considering the binary outcome of detecting a sound correspondence, we naturally set *p* = 0.5. In real language data, sound correspondence could be rare, so *p* will be much smaller. However, mathematically speaking, *p*(1−*p*) reaches its maximum value at *p* = 0.5, and the maximum value of *p*(1−*p*) leads to the maximum value of *n*, which makes Equation (8) equally applicable to cases having either many or few sound correspondences. In other words, the value *n* calculated at *p* = 0.5 is conventional, independent of potential sound correspondences in the exemplar set.

In Equation (8), the conventional size *n* relies on the total vocabulary size *N* of a language, and the significance level α and error rate ε of the sampling process. Here, we confine α and ε in a statistically acceptable range between 0.05 and 0.1, and set *N* within 4000–5000. Corpus linguists have estimated that this amount of words could cover more than 95% of the texts of a language (Laufer and Ravenhorst-Kalovski, 2010) and arguably suffice for basic comprehension (Nation and Warning, 1997).

We use Equation (8) to calculate the conventional size *n* in eight conditions formed by combinations of different values of ε (0.05 and 0.1), α (0.05 and 0.1), and *N* (4000 and 5000) (see the gray cells in Table 1).

In the strictest condition having the smaller error rate and significance level and the bigger total vocabulary size (ε = 0.05, α = 0.05 ($U_{\frac{\alpha }{2}}=1.96$), *N* = 5000), we have:

(9) $$\begin{array}{l} n=\frac{N{\left(U_{\frac{\alpha }{2}}\right)}^{2}p\left(1-p\right)}{N\varepsilon^{2}+{\left(U_{\frac{\alpha }{2}}\right)}^{2}p\left(1-p\right)}\\ =\frac{5000\;\times \;1.96^{2}\;\times \;0.5\;\times \;0.5}{5000\;\times \;0.05^{2}+1.96^{2}\;\times \;0.5\;\times \;0.5}=356.750\approx 357 \end{array}$$

In the most relaxed condition [ε = 0.1, α = 0.1 ($U_{\frac{\alpha }{2}}=1.645$), *N* = 4000], we have:

(10) $$\begin{array}{l} n=\frac{N{\left(U_{\frac{\alpha }{2}}\right)}^{2}p\left(1-p\right)}{N\varepsilon^{2}+{\left(U_{\frac{\alpha }{2}}\right)}^{2}p\left(1-p\right)}\\ =\frac{4000\;\times \;1.645^{2}\;\times \;0.5\;\times \;0.5}{4000\;\times \;0.1^{2}+1.645^{2}\;\times \;0.5\;\times \;0.5}=66.526\approx 67 \end{array}$$

**Figure 2:**
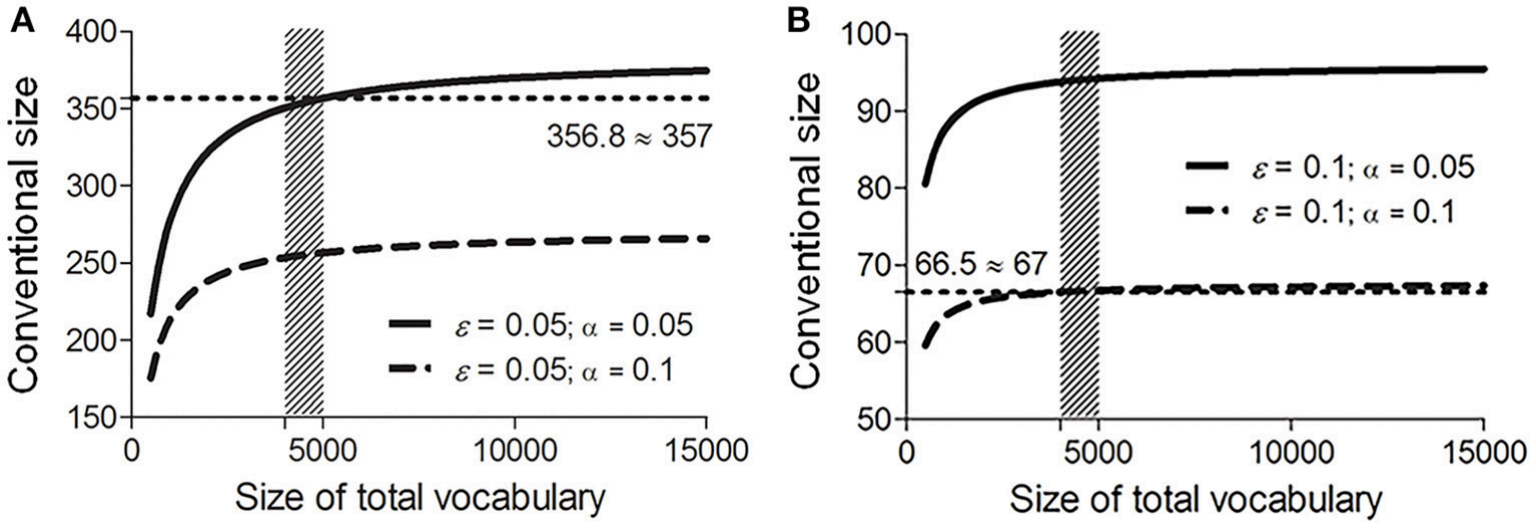
**Conventional sizes of the vocabulary list under fixed error rate ε [0.05 (A)** and 0.1 **(B)**] and significance level α [0.05 (solid lines) and 0.1 (dash lines)], and various total vocabulary sizes *N* (500–15000). Shade areas mark the range where *N* is between 4000 and 5000. Dotted lines mark the range of the conventional size *n* (round-up to the closest integers) calculated using Equation (8).

Conventional sizes calculated in the other six conditions all lie within the boundary values specified by Equations (9) and (10). Accordingly, the conventional size of the vocabulary list for assembling potential cognates is within the range [67, 357] (see Figure 2).

As shown in Figure 2 and Table 1, in a relaxed condition (ε = 0.1), when *N* is between 4000 and 5000, both sizes of the Swadesh lists (100 and 200) reach a significance level below 0.05 (α is within [0.02, 0.05] for size 100, and within [0.002, 0.01] for size 200). In other words, the distribution of sound correspondences in these amounts of words reliably reflects the distribution of those correspondences in the languages being compared (reaching a confidence level (1−α) above 95%; in statistical terms, in over 95% cases the distribution of sound correspondences in a sample of 100 or 200 words approximates, within the given error rate, the distribution of those correspondences in the total vocabulary set). These calculations indicate that both Swadesh lists are statistically large enough to estimate language relatedness.

**Figure 3:**
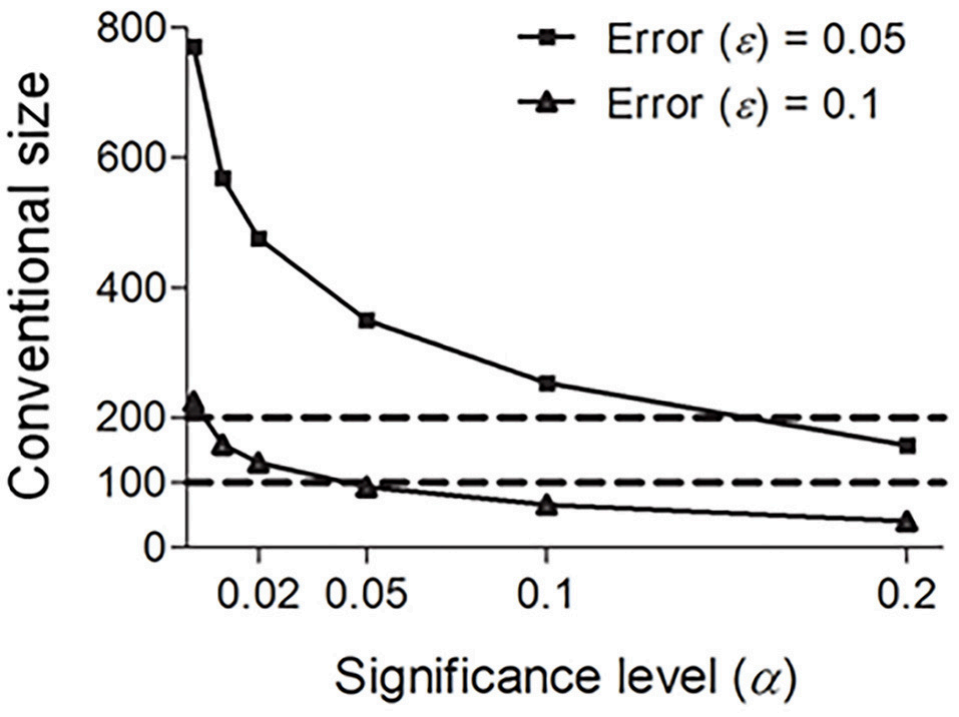
**Conventional size ***n*** under fixed error rates ε (0.05 and 0.1) and total vocabulary size ***N*** (4000), and various significance levels α**.

Some linguists (e.g., Embleton, 1986: p. 92–93) advocate using the Swadesh 200-word list rather than the Swadesh 100-word list because comparison accuracy decreases considerably when using a 100-word list. Our results suggest that the Swadesh 100-word list and the Swadesh 200-word list are both reliable, since their sizes lie in the conventional size range. Note that this does not mean that the two lists are interchangeable. In fact, they reach different significance levels (α). As shown in Figure 3, the Swadesh 200-word list shows a better performance than the Swadesh 100-word list; under the same sampling requirements (predefined significance level and error rate), the significance levels of the Swadesh 200-word list are consistently lower than those of the Swadesh 100-word list. By contrast, the reliabilities of the smaller lists containing 15, 33, 35, or 40 concepts are much lower (see Figure 3 and Table 1, under a total vocabulary of 4000–5000 words, the significance levels of those sizes are above 0.2, and their confidence levels are below 80%). In other words, these lists could not reliably reflect the distribution of sound correspondences in the languages being compared.

In addition, as shown in Figure 2, given fixed α and ε, the conventional size *n* increases steadily with the total vocabulary size *N*, yet such tendency becomes less explicit under a much bigger *N*. For example, in the most rigorous condition (α = 0.05, ε = 0.05; the solid line in Figure 2A), under a total of 15,000 words, the conventional size remains below 400. This indicates that a much bigger vocabulary list containing over 400 concepts offers no additional benefit in reflecting the distributions of sound correspondences in languages being compared. This is consistent with linguistic discussions (e.g., Ringe, 1992; Kessler, 2001). For example, Embleton points out that a word list having more than 500 items does not bring additional advantage in language comparison (Embleton, 1986).

Furthermore, since the above statistical analysis does not consider semantics, in principle, this conventional size range is instructive to cognate collection based on other linguistically acceptable meaning lists. Some linguists advocate using 300 concepts to sample potential cognates in Austronesian or Sino-Tibetan languages (e.g., Huang, 1997). This is because some concepts in the Swadesh lists (e.g., “bark,” “to swim,” “to lie,” or “because,” “in,” “at,” “with,” “if”) have no corresponding word forms in some Tibeto-Burman, Miao-Yao, or Zhuang-Dong languages (Jiang, 2007), whereas words encoding other concepts not in the Swadesh lists (e.g., “hemp,” “bamboo”) are arguably stable and resistant to borrowing (Li, 1995). Apart from these linguistic considerations, our quantitative analyses suggest that at least the size (300) of this vocabulary list is sufficient to collect potential cognates (see Figure 3 and Table 1, the confidence levels of size 300 under a total vocabulary of 4000 or 5000 words are above 90%).

### Generality of the Swadesh 100-word list

After proposing the Swadesh 200-word list in 1952, Swadesh published the 100-word list in 1955. He stressed that the 100-word list contained more stable concepts and the corresponding word forms tended to be less prone to borrowing (though the inherent stabilities of these words may differ, see Tadmor et al., 2010). Leaving aside linguistic considerations, after showing that both Swadesh lists are acceptable for cognate assembly, an immediate next question naturally arises: whether the Swadesh 100-word list is a special sub-list of the Swadesh 200-word list, in terms of the distribution of detected sound correspondences. Answer to this question is informative to cases where certain word forms in the Swadesh 100-word list do not exist or are generally hard to obtain. Such cases are common in reality (cf. Jiang, 2007).

Hypothesis testing in statistical inference serves as a useful means to address this question. In particular, the Ansari-Bradley test (Lunneborg, 2005) gives a 0–1 decision to the null hypothesis that two samples having common medians come from the same distribution. Decision “0” means that the null hypothesis cannot be rejected, and “1” that the null hypothesis can be rejected (that is to say, the two samples do not come from the same distribution) at a predefined significance level (say, 0.05). The Ansari-Bradley test is non-parametric and distribution-free; the samples for comparison do not need to have finite variances, identical sizes, or show normal distributions. Hence, this test is suitable for comparing the distributions of sound correspondences in assembled words, which follow binomial distributions. In addition, the Spearman's rank correlation coefficient (a.k.a Spearman's rho; Kornbrot, 2014) is another non-parametric measure of statistical correlation between two samples. It returns a value within [−1, 1], indicating the degree of negative or positive mutual dependence between any pairs of the data from the two samples. This measure is also suitable for revealing statistical dependence between sets of assembled words.

We discuss the generality of the Swadesh 100-word list based on the word forms assembled, respectively, in English and Latin according to the Swadesh 100- and 200-word lists (the data are extracted from Ringe, 1992; Table 2). For the sake of simplicity, we only consider sound correspondences appearing at fixed positions of assembled words. To be specific, we only consider the potential word-initial consonant correspondences shown in the assembled words.

**Table 2:** **Word-initial consonant correspondences (CCs) between English (left) and Latin (right) (extracted from Ringe, 1992) following the Swadesh 100- and 200-word lists**.

**Index**	**Concept**	**CC**	**Index**	**Concept**	**CC**	**Index**	**Concept**	**CC**
1	all(pl.)	∅-∅	49	leaf	l-f	99	you(sg.)	y-t
2	ashes	∅-k	50	lie	l-y	100	yellow	y-f
3	bark[of	b-k	51	liver	l-y	101	and	∅-∅
	tree]		52	long	l-l	102	animal	∅-∅
4	belly	b-w	53	louse	l-p	103	at	∅-∅
5	big	b-m	54	man	m-w	104	back[nn]	b-t
6	bird	b-∅	55	many	m-m	105	bad	b-m
7	bite	b-m	56	moon	m-l	106	because	b-k
8	black	b-∅	57	mountain	m-m	107	blow[vb,	b-f
9	blood	b-s	58	mouth	m-∅		wind]	
10	bone	b-∅	59	name	n-n	108	breathe	b-s
11	breast(s)	b-m	60	neck	n-k	109	child	c-p
12	burn[intr]	b-∅	61	new	n-n	110	count	k-n
13	claw	k-∅	62	night	n-n	111	cut	k-s
14	cloud	k-n	63	nose	n-n	112	day	d-d
15	cold	k-f	64	not	n-n	113	dig	d-f
16	come	k-w	65	one	w-∅	114	dirty	d-s
17	die	d-m	66	path	p-s	115	dull	d-h
18	dog	d-k	67	rain[nn]	r-p	116	dust	d-p
19	drink	d-b	68	red	r-r	117	fall	f-k
20	dry	d-s	69	root	r-r	118	far	f-p
21	ear	∅-∅	70	round	r-r	119	father	f-p
22	earth	∅-t	71	sand	s-h	120	few	f-p
23	eat	∅-∅	72	say	s-d	121	fight	f-p
24	egg	∅-∅	73	see	s-w	122	five	f-k
25	eye	∅-∅	74	seed	s-s	123	flow	f-f
26	fat[nn]	f-∅	75	sit	s-s	124	flower	f-f
27	feather	f-p	76	skin	s-k	125	fog	f-n
28	fire	f-∅	77	sleep	s-d	126	four	f-k
29	fish	f-p	78	small	s-p	127	freeze	f-g
30	flesh	f-k	79	smoke	s-f	128	fruit	f-p
31	fly[vb]	f-w	80	stand	s-s	129	grass	g-g
32	foot	f-p	81	star	s-s	130	guts	g-∅
33	full	f-p	82	stone	s-l	131	he	h-∅
34	give	g-d	83	sun	s-s	132	heavy	h-g
35	good	g-b	84	swim	s-n	133	here	h-h
36	green	g-w	85	tail	s-k	134	hit	h-f
37	hair[of	h-k	86	that(nt.)	*d*-∅	135	hold	h-t
	head]		87	this(nt.)	*d*-h	136	hunt[vb]	h-w
38	hand	h-m	88	tongue	t-l	137	husband	h-m
39	head	h-k	89	tooth	t-d	138	ice	∅-g
40	hear	h-∅	90	tree	t-∅	139	if	∅-s
41	heart	h-k	91	two	t-d	140	in	∅-∅
42	horn	h-k	92	walk	w-∅	141	knife	n-k
43	hot	h-k	93	water	w-∅	142	lake	l-l
44	human[nn]	h-h	94	we	w-n	143	laugh	l-r
45	I	∅-∅	95	what	w-k	144	left[-hand]	l-s
46	kill	k-∅	96	white	w-∅	145	mother	m-m
47	knee	n-g	97	who	h-k	146	narrow	n-∅
48	know	n-s	98	woman	w-m	147	near	n-p
148	now	n-n	165	sky	s-k	183	think	θ-k
149	old	∅-w	166	smell[tr]	s-∅	184	three	θ-t
150	other	∅-∅	167	smooth	s-l	185	throw	θ-y
151	play	p-l	168	snake	s-∅	186	tie	t-l
152	pull	p-t	169	snow	s-n	187	true	t-w
153	push	p-t	170	some(pl.)	s-∅	188	vomit	v-w
154	right[-hand]	r-d	171 172	spit split	s-s s-f	189 190	wash wet	w-l w-∅
155	river	r-f	173	squeeze	s-p	191	wide	w-l
156	rotten	r-p	174	stab	s-f	192	wife	w-∅
157	rub	r-f	175	stick[nn]	s-b	193	wind[nn]	w-w
158	salt	s-s	176	straight	s-r	194	wing	w-∅
159	scratch	s-s	177	suck	s-s	195	wipe	w-t
160	sea	s-m	178	swell	s-t	196	with	w-k
161	sew	s-s	179	there	*d*-∅	197	woods	w-s
162	sharp	s-∅	180	they	*d*-∅	198	worm	w-w
163	short	s-b	181	thick	θ-k	199	you(pl.)	y-w
164	sing	s-k	182	thin	θ-t	200	year	y-∅

*The first 100 concepts are from the Swadesh 100-word list. “∅” denotes zero consonant*.

Based on Spearman's rho, we compare the distribution of the sound correspondences in the assembled words following the Swadesh 100-word list with that in the assembled words following the Swadesh 200-word list. Spearman's rho in this test is 0.7384, with *p* < 0.0001. The *p* value indicates the chance for random sampling to reach the observed correlation if there is really no correlation between the two samples. The high Spearman's rho with an extremely low *p*-value reveals a high correlation between the distributions of the sound correspondences in the assembled words following the two Swadesh lists. This is also partially observed in Table 3. Based on the same setting and the statistical principle, identified recurrent correspondences based on the Swadesh 100-word list are largely consistent to those based on the Swadesh 200-word list.

**Table 3:** **Potential and recurrent word-initial consonant correspondences (CC) in the assembled words from English (left) and Latin (right) following the Swadesh 100- and 200-word lists (extracted from Ringe, 1992)**.

***A***	**Swadesh list**	**Potential CC**	**Recurrent CC**	**CC items**
0.01	100	62	6	∅-∅, f-p, h-k, l-y, n-n, r-r
	200	108	7	∅-∅, f-p, m-m, l-y, n-n, r-r, s-s
0.05	100	62	10	∅-∅, b-m, f-p, h-k, l-y, n-n, r-r, s-s, t-d, y-t
	200	108	15	∅-∅, b-m, f-p, h-k, l-y, m-m, n-n, p-t, r-r, s-s, ˘s-b, *d*-∅, θ-t, t-l, t-d

“*∅” denotes zero consonant*.

Based on the Ansari-Bradley test, we compare the distribution of sound correspondences in the Swadesh 100-word list with those in 10,000 100-word lists generated by random sampling from the Swadesh 200-word list. In these 10,000 comparisons, we count the total number of “1” decisions returned by the test. Such number is 11, corresponding to a *p*-value of 11/10,000 ≈ 0.0011. This indicates that in most situations the randomly sampled 100-word lists exhibit a similar distribution to the Swadesh 100-word list. In other words, the Swadesh 100-word list is not statistically distinct from other sub-lists of the same size.

Finally, we extend the above test by considering randomly generated sub-lists ranging in size from 20 to 200, incremented at a step of five words. For each list size *n*, we first sample a list of size *n* from the Swadesh-200 list, and then create another 10,000 sub-lists of *n* words. After that, we compare the distributions of the sound correspondences in the assembled words between the first list and each of the 10,000 sub-lists, and count the total number of “1” decisions returned by the test.

**Figure 4:**
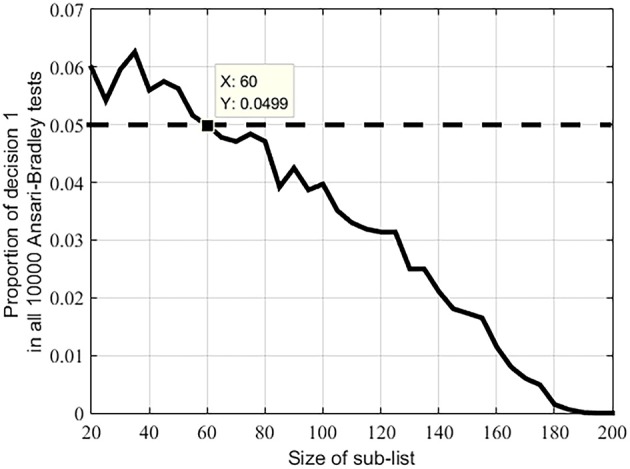
**Proportions of decision 1 in 10000 Ansari-Bradley tests under different sizes of sub-lists of the Swadesh 200-word list**. Dotted line indicates the significance level 0.05.

Figure 4 shows that in most randomly-created sub-lists containing 60 or more members the distributions of the sound correspondences are similar (at the predefined significance level of 0.05). This conclusion also holds for the Swadesh 100-word list; the distribution of the sound correspondences in any of the 100-word sub-list is similar.

These results indicate that although the meanings in the Swadesh 100-word list are carefully chosen according to linguistic considerations, the distribution of correspondences in the assembled words following this list is not significantly distinct from that following the Swadesh 200-word list or any 100-word sub-list of the Swadesh 200-word list. In other words, the Swadesh 100-word list is not statistically special.

The above tests verify the practice of using the Swadesh 100-word list in language comparison. Although some correspondence(s) may not exist in the assembled words following this list (see Table 3, which shows the potential and recurrent sound correspondences identified based on our second principle discussed in the next section), the findings remain statistically reliable. In addition, these tests also support the assumption that the semantics-phonetics mappings are largely arbitrary, such that word sampling is less confined by meanings. Furthermore, these tests give fieldwork linguists freedom to replace concepts in the Swadesh 100-word list that have no word forms with those in the Swadesh 200-word list (or other linguistically-acceptable list) that have word forms, and still obtain statistically similar results.

### Dynamic threshold of recurrent sound correspondence

As proved in Section Conventional Size of the Vocabulary List to Assemble Potential Cognates, if words for comparison are gathered following a vocabulary list having a conventional size, language affinity can be measured based on the number of detected recurrent sound correspondences in the collected words. Here, we revisit the statistical permutation principle (Ringe, 1992) to calculate the threshold for identifying recurrent sound correspondences among potential correspondences detected in assembled words. This principle is derived from the previous estimations of the degree of similarity that two (or more) languages are expected to show by chance (Bender, 1969; Oswalt, 1971; Ringe, 1993; Kessler, 2001). The logic behind the principle is as follows. If (1) a sound correspondence occurs in a few pairs of assembled words with semantic equivalence and (2) the accidental probability for such correspondence to randomly occur these many times is considerably low, the correspondence can be identified as a recurrent one. In addition, the minimum number of occurrences that keeps the accidental probability below a predefined significance level α can be assigned as the threshold for identification of recurrent correspondence.

Revisiting Figure 1, in the first step of lexicostatistics, the main concern is whether there are sound correspondences in the exemplars. Accordingly, we adopt the statistics of binomial distribution to calculate the conventional number of exemplars. In the second step, the focus shifts to counting the occurrences of a particular sound correspondence in the exemplars. In a Bernoulli process, the probability distribution of the number of occurrences follows a binomial distribution. Thus, based on the statistics of binomial distribution and the observed frequencies of relevant segments in the assembled words, we can calculate:

1. Accidental probability of a sound correspondence randomly occurring *n* times in the assembled words;
2. Minimum number of occurrence to keep the accidental probability of the sound correspondence below a considerably low level.

For two-language comparison, each occurrence of a sound correspondence (say, segment *a* in language A corresponds to segment *b* in language B) in the assembled words results from a combined Bernoulli process. The accidental probability *P* for such correspondence to occur randomly at least *k* times in the aligned words can be calculated as in Equation (11):

(11) $$\begin{array}{l} P\;(X\geq k)=1-\overset{k-1}{\underset{i=0}{\sum }}\left(\begin{array}{c} n\\ i \end{array}\right)p^{i}{\left(1-p\right)}^{n-i} \end{array}$$

Here, *n* is the number of assembled words, identical to the size of the vocabulary list used for collecting cognates, and *p* is the combined occurring frequency of the segments involved in this correspondence.

Without prior knowledge of the relations between languages being compared and considering the arbitrariness in mappings between semantics and phonetics, we can safely assume that the occurring frequencies of segments in these languages are independent, at least in the assembled words. Thus, the combined occurring frequency *p* of the segments in the assembled words can be calculated as the product of the occurring frequency of segment *a* in the assembled words from A (*p*_*A*_) and that of segment *b* in the assembled words from B (*p*_*B*_):

(12) $$\begin{array}{l} p=p_{A}p_{B} \end{array}$$

Note, that if there is a large degree of borrowing between the two languages, a dependent correlation between the occurring frequencies of segments in these languages can be easily observed.

**Figure 5:**
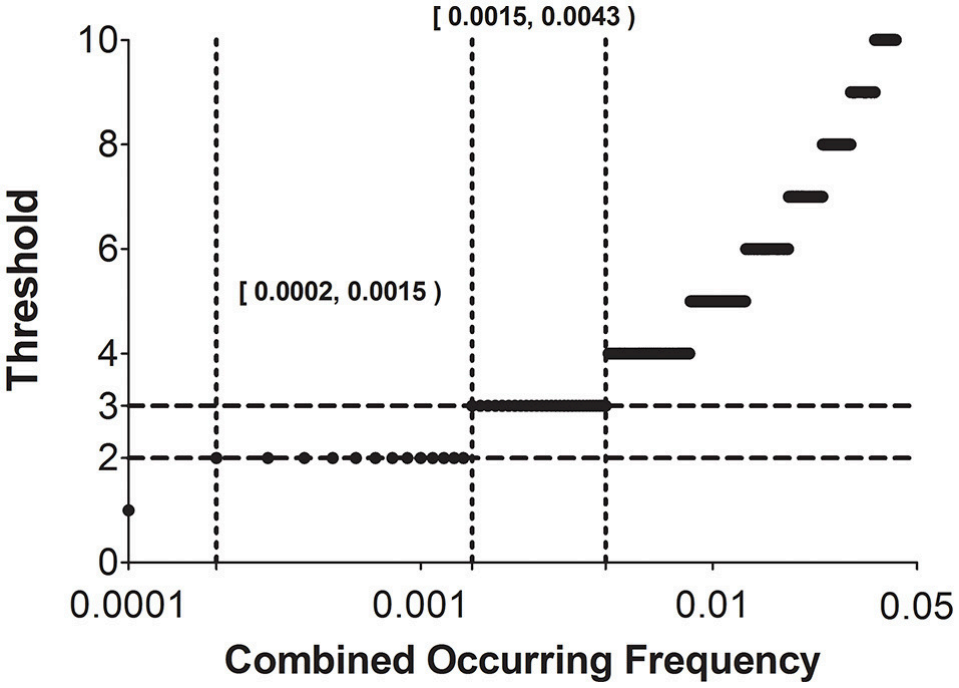
**Thresholds of recurrent sound correspondences under different combined occurring frequencies of involved sound segments (0.0001–0.05, with a step 0.0001)**. Two regions marked by dotted lines [0.0002, 0.0015) and [0.0015, 0.0043), respectively, denote the ranges of combined occurring frequencies where the threshold can be set to two and three.

Figure 5 traces the step-wise thresholds calculated by Equation (11) for two-language comparison using a Swadesh 100-word list. As shown in Figure 5, the smallest acceptable threshold is two (based on two matching instances). Compared to the other threshold values, the smallest threshold remains valid for the widest range of combined occurring frequency [0.0002, 0.0015). This indicates that the threshold two can reliably identify recurrent sound correspondences, provided that the combined occurring frequencies of these correspondences fall in this wide range. In addition, our principle suggests that the approach of using a fixed threshold throughout is not recommended. The threshold value must increase dynamically along with increase in combined occurring frequency.

Our principle (Equations 11, 12) enables an automatic assignment of threshold of recurrent sound correspondence. For example, if the combined occurring frequency of two segments falls in the range [0.0015, 0.0043), the threshold is set to three (according to Figure 5). Accordingly, if there are three or more pairs of the assembled words that recurrently show correspondence between the two segments, we can classify this correspondence as a recurrent one and those pairs of assembled words as cognates.

We use two examples that are much simpler than those in real cases to illustrate how to apply this principle. Using such simple examples avoids unnecessary distractions from linguistic aspects.

In the first example, the empirical data are word forms assembled, respectively, from English and French following the Swadesh 100-word list (extracted from Ringe, 1993; see Table 4). We apply our principle to calculate the accidental probability of a word-initial consonant correspondence /f/-/p/ in the assembled words.

**Table 4:** **Word-initial consonant correspondences (CCs) between English (left) and French (right) (extracted from Ringe, 1993)**.

**Index**	**Concept**	**CC**	**Index**	**Concept**	**CC**	**Index**	**Concept**	**CC**
1	all(pl.)	∅-t	34	good	g-b	67	path	p-s
2	ashes	∅-s	35	grease	g-g	68	rain[nn]	r-p
3	bark[of tree]	b-∅	36	green	g-v	69	red	r-r
4	belly	b-v	37	hair[of head]	h-s	70	root	r-r
5	big	b-g	38	hand	h-m	71	round	r-r
6	bird	b-∅	39	head	h-t	72	sand	s-s
7	bite	b-m	40	hear	h-∅	73	say	s-d
8	black	b-n	41	heart	h-k	74	see	s-v
9	blood	b-s	42	horn	h-k	75	seed	s-g
10	bone	b-∅	43	hot	h-s	76	sit	s-∅
11	breast(s)	b-s	44	human[nn]	h-p	77	skin	s-p
12	burn[intr]	b-b	45	I	∅-m	78	sleep	s-d
13	claw	k-g	46	kill	k-m	79	small	s-p
14	cloud	k-n	47	knee	n-z	80	smoke	s-f
15	cold	k-f	48	know	n-s	81	stand	s-d
16	come	k-v	49	leaf	l-f	82	star	s-∅
17	die	d-m	50	lie	l-∅	83	stone	s-p
18	dog	d-s	51	liver	l-f	84	sun	s-s
19	drink	d-b	52	long	l-l	85	swim	s-n
20	dry	d-s	53	louse	l-p	86	tail	s-k
21	ear	∅-∅	54	man	m-∅	87	that(nt.)	*d*-s
22	earth	∅-t	55	many	m-b	88	this(nt.)	*d*-s
23	eat	∅-m	56	meat	m-v	89	tongue	t-l
24	egg	∅-∅	57	moon	m-l	90	tooth	t-d
25	eye	∅-∅	58	mountain	m-m	91	tree	t-∅
26	feather	f-p	59	mouth	m-b	92	two	t-d
27	fire	f-f	60	name	n-n	93	water	w-∅
28	fish	f-p	61	neck	n-k	94	we	w-n
29	fly[vb]	f-v	62	new	n-n	95	what	w-k
30	foot	f-p	63	night	n-n	96	white	w-b
31	full	f-p	64	nose	n-n	97	who	h-k
32	give	g-d	65	not	n-n	98	woman	w-f
33	go	g-∅	66	one	w-∅	99	you(sg.)	y-t
						100	yellow	y-z

“*∅” denotes zero consonant*.

In this example, *p*_*A*_ is the occurring frequency of segment *a* (/f/) in the assembled words from English, and *p*_*B*_ is that of segment *b* (/p/) in the assembled words from French. In the assembled words from English, the word-initial consonant /f/ appears ten times; in those from French, the word-initial consonant /p/ appears six times. Following Equation (12), the combined occurring frequency *p* of the two segments is 0.006(= 0.1×0.06). Following Equation (11), the accidental probability *P* for the correspondence /f/-/p/ to occur randomly at least four times is:

(13) $$\begin{array}{l} P\left(X\geq 4\right)=1-\overset{3}{\underset{i=0}{\sum }}\left(\begin{array}{c} 100\\ i \end{array}\right)0.006^{i}{\left(1-0.006\right)}^{100-i}\\ =1-\left(\begin{array}{c} 100\\ 0 \end{array}\right)0.006^{0}{\left(1-0.006\right)}^{100}\\ -\left(\begin{array}{c} 100\\ 1 \end{array}\right)0.006^{1}{\left(1-0.006\right)}^{99}\\ -\left(\begin{array}{c} 100\\ 2 \end{array}\right)0.006^{2}{\left(1-0.006\right)}^{98}\\ -\left(\begin{array}{c} 100\\ 3 \end{array}\right)0.006^{3}{\left(1-0.006\right)}^{97}\\ =0.0032 \end{array}$$

Statistically speaking, the accidental probability 0.0032 is much lower than the general significance level α (0.01 or 0.05), and among 100 pairs of the assembled words, there are four pairs that exhibit such correspondence (i.e., father vs. *père*, fish vs. *poisson*, foot vs. *pied*, and full vs. *plein*). Therefore, we can safely claim that the correspondence /f/-/p/ is recurrent and those four pairs of words are cognates. Similarly, as shown in Figure 5, since its combined occurring frequency is 0.006, the threshold for classifying it as a recurrent one should be four.

In the second example, the empirical data are word forms assembled, respectively, in Latin and English according to the Swadesh 100− and 200-word lists (extracted from Ringe, 1992; see Table 2). We use our principle to evaluate all detected word-initial consonant correspondences (see Table 3), under two significance levels, 0.01 and 0.05.

At the significance level 0.01, in the words assembled following the Swadesh 100-word list, we detect 62 correspondences. Based on the occurring frequencies of related consonants in the assembled words and Figure 5, our principle identifies six recurrent correspondences, matching exactly those identified by Ringe (1992). At the same significance level, in the words assembled following the Swadesh 200-word list, we detect 108 correspondences. Based on the occurring frequencies of related consonants and Equations (11) (set *n* = 200) and (12), our principle identifies seven recurrent correspondences. There are differences between the two sets of recurrent correspondences obtained following the two lists: /h/-/k/ in the Swadesh 100-word list, whereas /m/-/m/ and /s/-/s/ in the Swadesh 200-word list. At the significance level 0.05, our principle identifies more recurrent correspondences (10 in the Swadesh 100-word list and 15 in the Swadesh 200-word list).

The above examples were first used by Ringe (1992, 1993), who attempted to show that no matter what size the vocabulary list has the relative frequency between related and unrelated words remained the same and the numbers of matches expected by chance were proportional to the number of words (Kessler, 2001). Our results demonstrate two things: (1) under the same vocabulary list, different significance levels lead to identification of different sets of recurrent sound correspondences and (2) under the same significance level, size differences in vocabulary lists also lead to differential identification outcomes. These results suggest that our principle is dependent on a number of numerical parameters, including the size of the vocabulary list for word assembly, the occurring frequencies of related sound segments in the assembled words, and the predefined significance level.

In addition, if one sticks to the smallest threshold (two), some correspondences would be incorrectly classified as recurrent. For example, in the assembled words following either list, the correspondences /b/-∅ and /f/-∅ occur four and two times, respectively. Based on the threshold two, both correspondences would be deemed as recurrent. However, linguists have proved that neither of them is recurrent. By contrast, according to our principle, due to their high combined occurring frequencies, the threshold of these correspondences should be set as a value much bigger than their occurring numbers in the assembled words. Then, in line with linguistic considerations, both correspondences are not judged as recurrent.

## Discussion

In lexicostatistics, linguistic intuitions and subjective experiences have been the primary factors determining (1) which words should be collected for comparison, (2) how many words are needed for comparison, and (3) whether a specific number of matching instances is sufficient to confirm a “recurrent” correspondence for identifying cognates (Hock and Joseph, 1996; Baxter and Ramer, 2000; Brown et al., 2013). We use statistical principles to independently validate the reliability and generality of the results pulled from the commonly used Swadesh 100- and 200-word lists. We also use statistical theorems to provide objective answers to questions (2) and (3): we propose a method to quantify the conventional size of vocabulary lists for word assembly, as well as a method to assign reasonable thresholds for detecting recurrent sound correspondences. Our results show that (1) the widely-adopted practice of using 100 or 200 words following the Swadesh lists for cognate assembly can render reliable comparison; (2) the Swadesh 100-word list is statistically invariant to the Swadesh 200-word list and other sub-lists having comparable sizes; and (3) the threshold based on at least two matching instances remain valid for a wide range of cases, yet such threshold must increase dynamically with increase in combined occurring frequencies of relevant sound segments.

Our study reveals that in addition to linguistic considerations, statistical criteria (e.g., significance level and error rate) are also critical for comparison outcome. Based on different sampling requirements, the same datasets may render distinct results. Therefore, respecting and applying statistical criteria is necessary and beneficial to verify, replicate, and discuss language comparison studies based on the same datasets. As social scientists, linguists generally receive less mathematical training in developing or evaluating statistical approaches (Baxter and Ramer, 2000). Nonetheless, their linguistic intuitions and rich experiences turn out to be reliable to a certain extent to bring forth informative understanding about historical relations among languages. With more and more large-scale datasets being available, our study can guide future research based on such datasets.

Identifying recurrent sound correspondence is an important step not only in lexicostatistics to detect cognates, but also in comparative method to reconstruct linguistic features of protolanguage. Among the available probability approaches adopted to do this task, such as Chi-square calculations (Ross, 1950; Kessler, 2001), Binomial approach (Ringe, 1992) and Shift test (Oswalt, 1971), our statistical principle follows Ringe's Binomial approach. This approach explicitly assigns the meaning to the parameter *p* (probability for two segments to exhibit a sound correspondence). In addition, the Binomial approach can also be efficiently applied in the cases where there are very low expected numbers in the slots of the table for comparison. In such cases, Chi-Squared test cannot be used (McMahon and McMahon, 2005). Note that despite of its advantages, some linguists criticize that the Binomial approach could be too rigorous to confirm close relationship within Indo-European languages (Greenberg, 1993: p. 89). Furthermore, the rationale of our method is similar to that of Bayesian approach. In principle, Bayesian approach is to estimate a posterior probability with respect to a prior probability based on the given data. It depends excessively on the prior distribution, and determination of this distribution is subject to the intuition and experience of researchers. In our study, the frequencies of the segments occurring in a given word list can be treated as the prior distribution. Whether a correspondence is recurrent or not cannot be simply determined by the occurrence of such correspondence. Instead, it needs to take into consideration not only the number of occurrence of the correspondence but also the number of occurrence of the segments involved in the correspondence, the latter of which could be biased due to word collection.

Mathematicians and statistical physicists have developed powerful approaches, many of which have potential applications in linguistics. Our study attempts to bridge the gap between linguistics and other disciplines, by exemplifying how to employ statistical knowledge and approaches to tackle linguistic issues. There have been more and more attempts like this (e.g., Bouchard-Côté et al., 2013; Hruschka et al., 2015). Integration of multidisciplinary approaches has become imperative to evaluate data collection and classification methods widely-adopted in linguistics and other social science disciplines.

## Author contributions

MZ and TG designed the research, MZ carried out the study. MZ and TG analyzed the results. MZ and TG wrote the paper.

### Conflict of interest statement

The authors declare that the research was conducted in the absence of any commercial or financial relationships that could be construed as a potential conflict of interest.
